# Microencapsulated algal feeds as a sustainable replacement diet for broodstock in commercial bivalve aquaculture

**DOI:** 10.1038/s41598-020-69645-0

**Published:** 2020-07-31

**Authors:** David F. Willer, Samuel Furse, David C. Aldridge

**Affiliations:** 1 Department of Zoology, The David Attenborough Building, University of Cambridge, Pembroke Street, Cambridge, CB2 3QZ UK; 2 Institute of Metabolic Science, Addenbrookes Hospital, Cambridge, CB2 0QQ UK

**Keywords:** Biotechnology, Marine biology

## Abstract

The global bivalve shellfish industry makes up 25% of aquaculture, is worth USD $17.2 billion year^−1^, and relies upon a supply of juvenile bivalves produced by adult broodstock in hatcheries. Today large quantities of live algae are grown to feed broodstock at $220 kg^−1^, driving highly unsustainable energy and resource use. New advances in algal and microencapsulation technology provide solutions. We developed microencapsulated *Schizochytrium* algae diets, which can be produced sustainably at < $2 kg^−1^ from organic side-streams, and are shelf-stable to minimise waste. Physiological, histological, and cutting-edge metabolomic analyses demonstrate that in commercial settings sustainable microencapsulated diets facilitate improved sexual development and 12 × greater omega-3 levels in oysters relative to conventional live algal diets. Every tonne bivalve protein produced instead of fish spares 9 ha, 67 tonnes CO_2_, and 40,000 L freshwater. Further research into microencapsulated diets could support bivalve industry expansion, and contribute towards a step-change in sustainable global food production through improved aquaculture practices.

## Introduction

The USD $17.2 billion global bivalve shellfish industry relies upon a supply of juvenile bivalves produced by adult broodstock in hatcheries^1^. Current estimates suggest 220 million broodstock bivalves are held in hatcheries worldwide^1–6^. At present broodstock must be fed live algae, production of which drives unsustainable land, energy and antibiotic use^6^.

Algal production in hatcheries makes highly inefficient use of land and energy^6–8^. Typical hatcheries require 400 m^2^ of algal tanks to maintain just 400 broodstock, equating to 220 million m^2^ worldwide, an area larger than Washington, D.C.^4^. High-intensity artificial lighting, temperature, and air control systems are needed to support algal growth^9^. Furthermore, algal stocks are difficult to maintain and frequently lost due to contamination and disease, so greater quantities must be produced, to the extent that each unit of viable hatchery-grown algae is 20-fold more expensive than units grown in contamination-free commercial photobioreactor facilities^6,10–12^, accounting for 50% of bivalve production costs at USD $220 kg^−1^ algal biomass in 2016^13,14^.

Algal production presents a major disease control issue. Live algal feeds are the primary vector of bivalve disease, which is controlled with antibiotics^6^. Antibiotics cause severe damage to marine ecosystems, 80% of aquaculture antibiotics are not metabolised by stock, persist in the open sea, and drive proliferation of antibiotic resistant bacteria^15,16^. In the world's largest bivalve producers including China no veterinary prescriptions are required for antibiotics with use essentially unregulated^15^. In Europe twice the number of antimicrobial substances were sold for animal versus human use in 2014^15^. Non-live diets are more sterile, and hence since the 1990s the bivalve aquaculture industry has been seeking non-live alternative feeds to reduce antibiotic needs^6,7,11,16^. FAO and EU sustainable aquaculture policies identify urgent and immediate needs to reduce land, energy, and antibiotic use in aquaculture^15,17–19^.

New advances in algal production and microencapsulation technology offer a groundbreaking solution to reduce the environmental footprint of bivalve aquaculture. *Schizochytrium* algae can be grown heterotrophically on industrial scales at USD $1.50 kg^−1^, using low-cost food waste and agricultural side-streams as inputs^12,20–22^. For bivalve nutrition *Schizochytrium* has advantages, with levels of key nutrients such as docosahexaenoic acid (DHA) exceeding 20% dry-weight; greater than twice the abundance of DHA in hatchery-grown algae^22,23^. Novel microcapsules are an ideal vehicle for delivering sustainable *Schizochytrium*-based diets to bivalve broodstock^6,8,11,24^. Mass production is simple and cost-effective^8,24^, and the microcapsules dry and shelf-stable thus circumventing conventional feed wastage costs^16^. Capsule characteristics can be tailored to maximise feeding efficiency^16^ and minimize nutrient leaching to water^7,25,26^, whilst also being sterile and not a disease vector^6^. The nutritional profile of microencapsulated feed compared to conventional algal feed is shown in Table 1.

**Table 1:** Nutritional composition of broodstock diets: live algae, microcapsules, or live algae + microcapsules.

Diet	Protein	Carbohydrate	Lipid	Ash and fibre	20:5n-3 (EPA)	22:6n-3 (DHA)
Live algae	31	12	13	44	2.31	0.70
Microcapsules	6	16	58	20	3.25	9.01
Live algae + microcapsules	18	14	36	32	2.78	4.85

All values are in g per 100 g dry weight (dw). Nutritional data is sourced directly from the manufacturer—for live algae SeaSalter Shellfish (Whistable) Ltd, for microcapsules BioBullets Ltd, and for the combined diet a mean is used.

**Figure 1:**
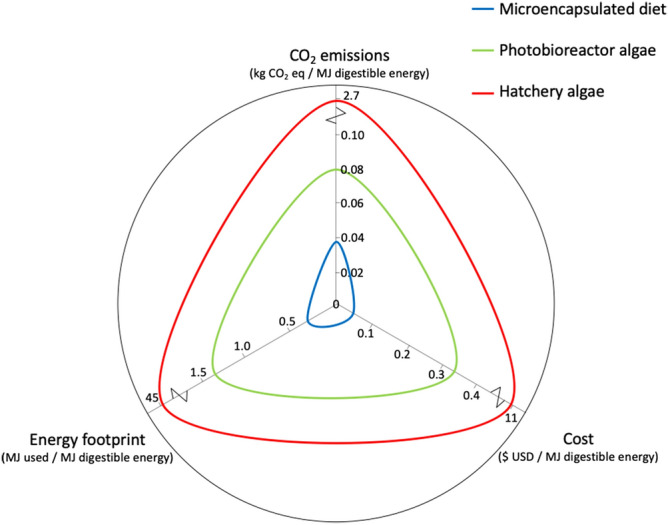
The sustainability advantage of new microencapsulated diets. The radar-plot demonstrates lower CO_2_ emissions, reduced energy usage, and more efficient use of economic resources in new microencapsulated diets. Comparison is relative to the most efficient to produce form of autotrophic live algae grown on an industrial scale (photobioreactor algae), and algae grown in a relatively efficient bivalve hatchery today. Note the broken axes for hatchery algae. We also note that a 100% replacement of live algae will require further tailoring of the microcapsule formulation for increased protein content. Data sources and figure methodology: Supplementary Data S1^8,11–14,20,24,27–30^.

There are major sustainability advantages of replacing live algal feeds with microencapsulated feeds in bivalve aquaculture, including 20-fold reductions in energy use, carbon emissions and production costs (Fig. 1). However, for replacement to be commercially viable it is critical to assess whether microencapsulated feeds provide comparable sexual development in bivalve broodstock compared to conventional algal feeds.

## Results

### Sexual maturation: gonad weight

**Figure 2:**
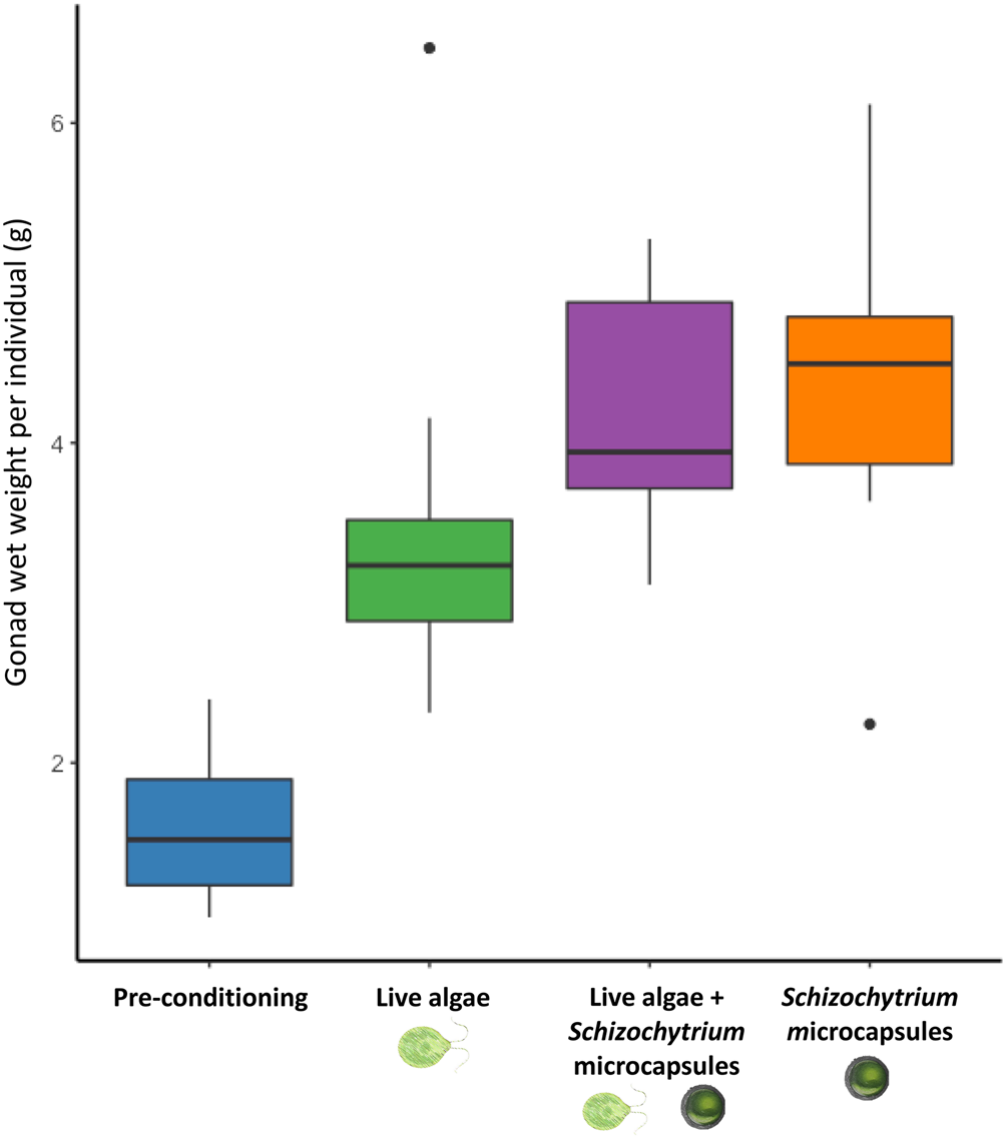
Increased oyster broodstock gonad weight following conditioning. The boxplot shows the total wet gonad weight of oysters fed algae (green), microcapsules (orange), or algae + microcapsules (purple) over a 6-week conditioning period, relative to oysters pre-conditioning (blue). n = 9 oysters per diet, gonad weight was significantly greater in oysters fed a diet relative to oysters pre-conditioning.

We first tested the impact of replacing live algal diets with microencapsulated *Scizochytrium* diets on oyster gonad weight. Both microencapsulated and live algal diets resulted in greater gonad weight European oysters (*O. edulis*) following a 6-week broodstock conditioning period in a commercial hatchery, relative to pre-conditioning control (t = 0) oysters. Mean wet gonad weight was significantly greater in oysters fed algae (3.51 ± 1.24 g (Standard Error)), microcapsules (4.40 ± 1.10 g), or algae + microcapsules (4.15 ± 0.75 g) compared to oysters pre-conditioning (1.59 ± 0.46 g) (ANOVA, F_3,32_ = 16.58, p < 0.001). Figure 2 demonstrates how the difference between sample and control gonad weight was greatest in oysters fed microcapsules (2.81 ± 1.10 g), although this value was not significantly different from oysters fed algae (1.92 ± 1.24 g) (ANOVA, F_2,24_ = 1.73, p = 0.19). The observed greater gonad weight in conditioned relative to pre-conditioned oysters is expected as oysters build up energy reserves for gametogenesis, and the greater values can be assumed to represent an increase in weight over time relative to the controls^31^.

### Sexual maturation: fatty acid and lipid abundance

Demonstration that microencapsulated *Schizochytrium* diets could facilitate comparable or greater increases in gonad mass compared to live algal diets provided macroscopic evidence that microencapsulated diets could be an effective replacement for live algae in sexual maturation. We hence embarked upon a molecular investigation of gonad lipids and gametogenesis to provide further explanation.

**Figure 3:**
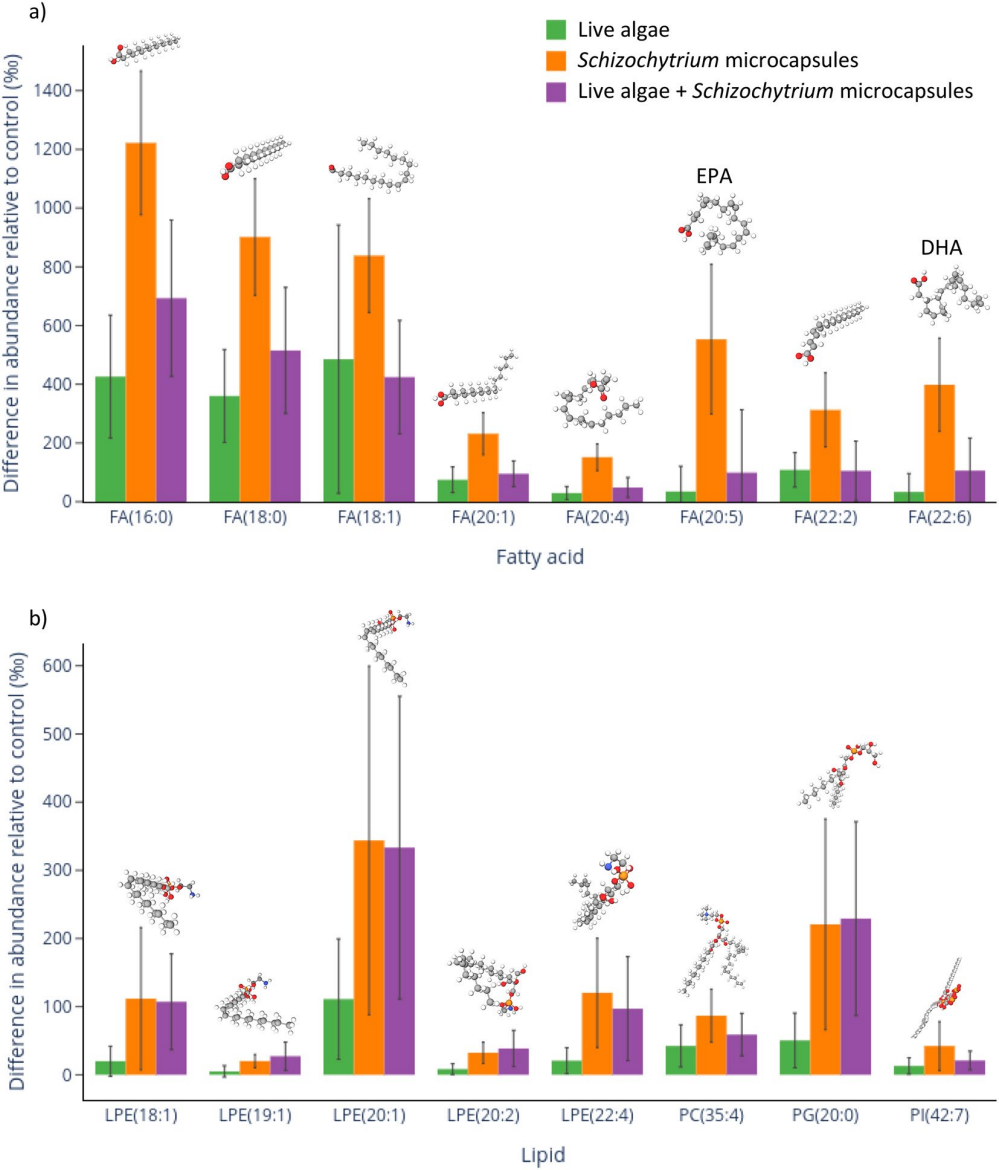
Increase in abundance of fatty acids (**a**) and other lipids (**b**) over conditioning period. The increase shown in the bar plots represents the difference in fatty acid and lipid abundance in the gonads of oysters fed algae (green), microcapsules (orange), or algae + microcapsules (purple) over a 6-week conditioning period, relative to oysters pre-conditioning. Abundance was calculated from mass spectrometry data using total wet gonad weight as a scaling factor. n = 9 oysters per diet, error bars represent standard error. The eight fatty acids (**a**) and lipids (**b**) with the greatest change in abundance across all diets are presented in the bar plots, and for these all diets differ significantly (p < 0.05 with Holm-Bonferroni). For (**b**) lipid names have been abbreviated for clarity, full names are shown in Table S2.

Mass spectrometry was used to determine abundance and profile of fatty acids. These data showed that fatty acid mass in *O. edulis* gonads was greater post-conditioning compared to pre-conditioning. The greatest differences in abundance (over 400 ‰) were present in 16:0, 18:0, 18:1, 20:5 (EPA), and 22:6 (DHA) fatty acids (Fig. 3a). There was a significant difference between diets in the magnitude of the abundance difference for 42 of the 45 fatty acids analysed (see Supplementary Information Table S1a).

The greatest difference in fatty acid abundance relative to the pre-conditioning controls was seen in oysters fed only microcapsules. For 40 of the 45 fatty acids the difference was significantly greater for microcapsule fed oysters compared to algae fed oysters (Table S1a). In particular, the difference in 20:5 and 22:6 fatty acids was 12 times greater in microcapsule compared to algae fed oysters (microcapsule fed: 20:5 = 553.3 ± 254.8 ‰, 22:6 = 398.5 ± 158.6 ‰; algae fed: 20:5 = 34.9 ± 86.0 ‰, 22:6 = 33.4 ± 62.6 ‰). The post-conditioning difference in fatty acid abundance was also significantly greater in oysters fed only microcapsules compared to oysters fed algae + microcapsules in 41 of 45 cases (Table S1a). Whilst algae + microcapsule fed oysters did in general have greater post-conditioning differences in fatty acid abundance than algae fed oysters, this difference was only statistically significant in 3 cases (Table S1a).

The abundance of other lipids in *O. edulis* gonads was also greater post-conditioning compared to pre-conditioning, although there was only a significant difference between diets for 30 of the 792 lipids assessed. Table S1b presents lipids where there was a significant difference between diets; the remainder of the lipids can be found in Table S2. For the following four lipids the difference in abundance was particularly large (over 100 ‰) and also significantly greater in oysters fed microcapsules or algae + microcapsules compared to oysters fed algae alone: LPE(18:1)_[M-H]1-, LPE(20:1)_[M-H]1-, LPE(22:4)_[M-H]1-, PG(20:0) (Fig. 3b, Table S1b).

The significantly greater post-conditioning abundance of fatty acids and lipids in oysters fed microcapsules provide a biochemical explanation to our initial finding of strong increases in gonad mass in oysters fed microcapsules. We can again assume that the greater abundance represents an increase over time relative to the pre-conditioning controls^31^.

### Sexual maturation: histological imaging

**Figure 4:**
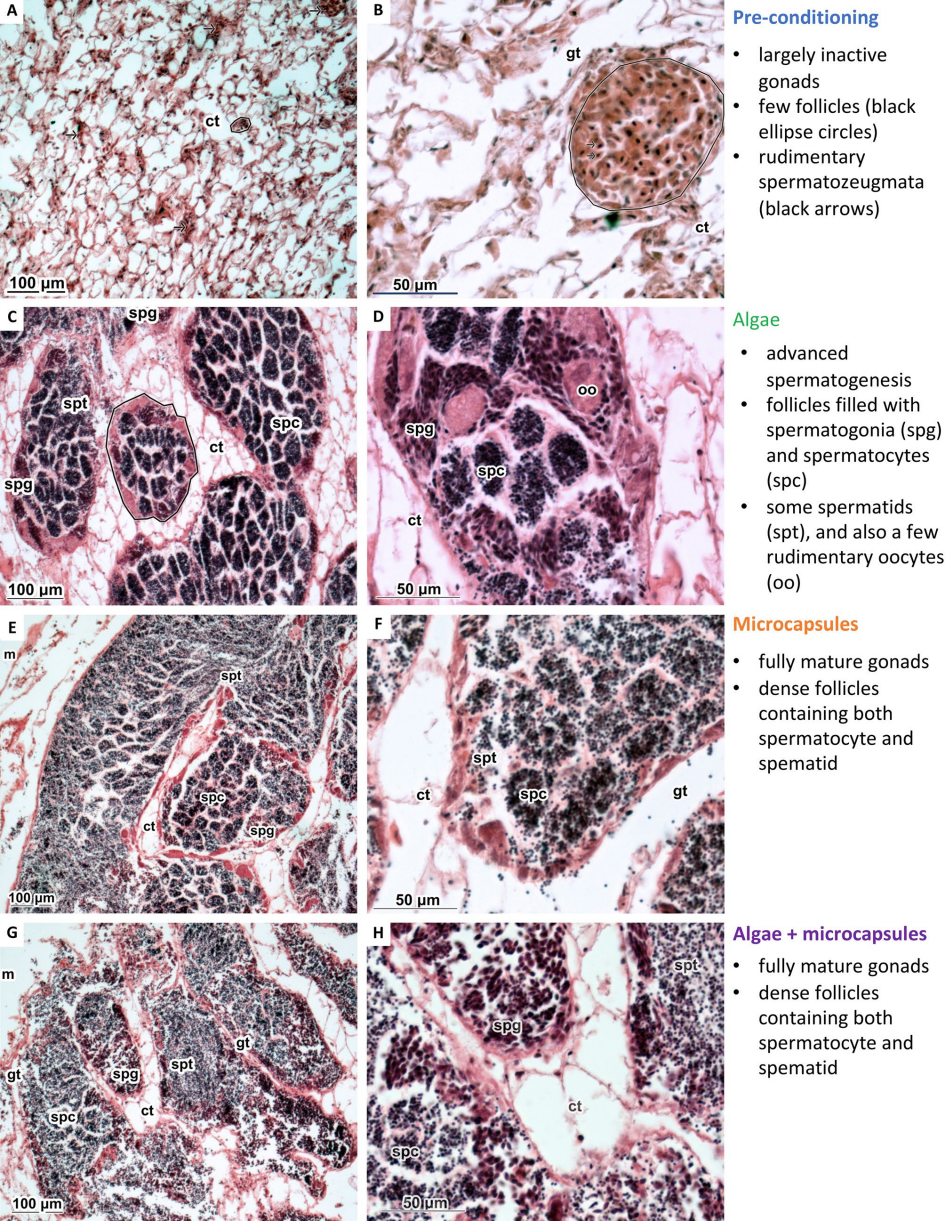
Greater sexual maturation in oysters fed *Schizochytrium* microcapsules. Histological sections of *O. edulis* gonads from predominantly male individuals fed one of three diets compared to pre-conditioning controls: pre-conditioning (**a**,**b**), algae (**c**,**d**), microcapsules (**e**,**f**), algae + microcapsules (**g**,**h**). Selected images are haematoxylin eosin stained and represent the typical gonadal state of five oysters from each treatment type after a 6-week feeding period. ct: connective tissues; gt: gonadal tubule; m: mantle.

Histological imaging of the oyster gonads revealed that oysters fed either microcapsules or algae + microcapsules were at a more advanced stage of sexual maturation after 6-weeks of conditioning than oysters fed algae alone (Fig. 4). Oysters fed algae alone had progressed from having largely inactive gonads (Fig. 4a,b) to advanced spermatogenesis, with follicles filled with spermatogonia and spermatocytes (Fig. 4c,d). In contrast, oysters fed microcapsules in addition to or in replacement of algae had reached full maturity and had dense follicles with many spermatids (Fig. 4e–h).

## Discussion

### Microencapsulated diets enable improved sexual maturation in oysters

Our investigations demonstrate that *Schizochytrium*-based microencapsulated diets enable not only comparable but improved sexual maturation in oyster broodstock compared with conventional live algal diets. The gonads of oysters fed microencapsulated diets were of greater weight, contained higher levels of omega-3 fatty acids crucial for sexual maturation, and underwent accelerated spermatogenesis.

Of particular importance are the large increases in EPA and DHA in microcapsule relative to algae fed oysters. EPA is the primary energy source for gamete maturation, with higher levels directly increasing gamete quantity and development rate^32^. DHA is pivotal to the structure and function of gamete cell membranes, with higher levels increasing gamete quality and egg survival rates^31–35^. High levels of EPA and DHA in the *Schizochytrium*-based microcapsules are likely driving this increase^22,23^. To date there is no clear evidence that either fatty acid can be synthesised de-novo by oysters from shorter chain precursors^32,36^. The more rapid advance of spermatogenesis in oysters fed a microencapsulated diet is highly likely being driven by the greater levels of EPA and DHA in these animals,a causal relationship demonstrated by several previous studies^9,37,38^. This offers strong support for the use of microcapsules as a broodstock conditioning feed.

It is important to consider that for future application the nutritional formulation of the microcapsules would need to be tailored further for increased protein content or fed alongside a quantity of live algae. The current protein content of the microcapsules is lower than that of live algae (6 vs. 31 g protein per 100 g dry weight, Table 1). Protein is important in bivalve larval development and for shell formation, and if insufficient juvenile growth can be suppressed^11^. There would be significant value in performing additional studies investigating the effectiveness of a higher protein formulation of microcapsules on bivalve broodstock conditioning and juvenile development.

Regarding the newly developed microcapsules; the size is tailored to maximise bivalve feeding efficiency (20–140 µm diameter), and buoyancy neutral to ensure particles remain within reach of the filter feeders^16^. This is an improvement over a basic freeze-dried algal powder delivery system,powders tend to float on the water surface, and can clump into particles too large to be accessed by bivalves^7^. The waxy encapsulant minimises pre-ingestive nutrient loss by allowing particles to remain stable and retain nutrients in seawater, yet still be rapidly digested on entry to the bivalve gut^7^. The specialised coating allows delivery of low molecular weight, water soluble compounds, alongside fatty acids, with minimal leaching to the surrounding water^25^^,^ reducing eutrophication risks^7,26^. The encapsulant also has strong antibacterial properties, and contents are sterile, which reduces disease incidence in aquaculture relative to live feeds^11,39^, and enables reduced antibiotic usage^39^. There remains scope for further experiments allow greater optimization of the physical characteristics of the microcapsules. For example, whilst we know that capsules of 20–140 µm can be ingested by oysters^40^^,^ it would be useful to perform a study to investigate the filtration and ingestion of different sizes of microcapsule within this range to identify the preferred specific capsule diameters for both bivalve broodstock and juveniles.

### Sustainability and commercial implications

The use of live algae is driving excessive and unsustainable energy and resource use in bivalve production. This investigation demonstrated that sustainable *Schizochytrium*-based microencapsulated diets can help support more effective sexual maturation in oyster broodstock. Further research could help open up the opportunity initially outlined; to upscale production and associated infrastructure to allow microencapsulated diets to partially or fully replace live algae in hatcheries, and reduce the environmental footprint of bivalve aquaculture.

However, given that microencapsulated diets enabled not only comparable but improved sexual development in bivalve broodstock, there is potential to reap even further commercial and sustainability benefits. Higher quality broodstock with greater lipid stores directly translates into higher quality seed with a greater inherent survival rate^32^. More rapid sexual maturation enables seed production earlier in the season, giving the seed a greater growing period prior to their first overwintering, with the corresponding greater size and cold-tolerance again increasing survival^9,41^. Increased sexual maturation rates also mean shorter conditioning cycles and greater larvae production for a given hatchery each year, increasing the total output of bivalve seed^9^. As the supply of bivalve seed is one of the biggest factors limiting the growth of the bivalve industry, with demand far outstripping supply, microencapsulated feeds could play an important role in enabling the bivalve industry to expand^9,11,41^. Key next steps towards reaching this goal will include further tailoring of the microcapsule formation for increased protein content and use-specific size profile to maximise ingestion efficiency and bivalve growth.

Bivalve aquaculture is far more environmentally sustainable than other forms of aquaculture and meat production, and even some cereal crops^6,19^. For every new tonne of protein that is produced from bivalve instead of fish aquaculture, we spare 9 ha land, 67 tonnes CO_2_ emissions, and 40,000 L freshwater^6^. Any technology, such as microencapsulated diets, that might enable bivalve aquaculture to grow instead of other aquaculture should be viewed as of great benefit and a worthwhile recipient of further research and industry attention. More widely, live algae remains a key component in the USD $99.3 billion fish aquaculture industry, where application of microencapsulated diets would also be appropriate^16,25,42^. New microencapsulated diets derived from aquacultural waste streams could contribute towards a step change in sustainable global food production through improved industry practices.

## Methods

### Microcapsule manufacture

Lipid-walled microcapsules containing 50% powdered *Schizochytrium* algae by weight were manufactured under patent by BioBullets (BioBullets Ltd, Cambridge, UK). To manufacture the particles a premix slurry containing a waxy encapsulant with antibacterial properties and powdered algae were prepared under conditions of controlled shear. The slurry was pumped into an ultrasonic atomizing nozzle at the top of a cooling chamber. The atomized particles formed near-perfect spheres as they cooled and fell to the chamber base. Further particle cooling was achieved with an air-conveying system before discharge via cyclone to a fluid bed processor. The encapsulated particles were then coated with a proprietary non-ionic surfactant to aid dispersion in water. Further cooling in the fluid bed removed all heat of crystallization from the microparticles before packaging^8^. All components of the formulation were food grade. The final microcapsules had a diameter between 20 and 140 µm, spherical shape, and near neutral buoyancy.

### Broodstock conditioning

Conditioning experiments on *O. edulis* broodstock took place under commercial production conditions at SeaSalter Shellfish (Whistable) Ltd, Kent, England. Experiments took place over 6-weeks between 29/03/2018 and 10/05/2018. These were carried out in three 25 L aerated flow-through tanks kept at ambient hatchery temperatures (18–24 °C) and salinities (26–28 ‰)—offering further commercial context to our experiments although presenting a limitation in the form of the necessity for pseudoreplication. Each tank contained 15 *O. edulis* broodstock and received one of the following three diets: live algae (SeaSalter's formulation), microcapsules (BioBullets), or algae + microcapsules. The nutritional profiles of each diet were obtained from the manufacturer and are shown in Table 1. Each feed was fed at 3% g dw feed g mean dw broodstock^−1^ day^−1^, meaning oysters on the algae + microcapsules diet received twice as much food as oysters on the single food diets. The 3% ration is recommended by SeaSalter and additional algal ration above this value has been shown to have little effect on *O. edulis* nutrient uptake or growth^9^^,^ ensuring that any differences in growth when microcapsules were added would likely be driven by improved nutritional value rather than increased ration. Feed was delivered using a continuous system with a flow-through rate of 10 ml min^−1^, with the feed lines discharging halfway to the base of each tank. At the end of the 6-week conditioning period all broodstock were frozen and transported in cool boxes to the Department of Zoology, University of Cambridge, England, where they were then frozen at − 80 °C. Before the conditioning period began an additional sample of 15 broodstock was also transported to Cambridge and frozen at − 80 °C for use as pre-conditioning controls.

### Gonad weight analysis

The entire gonad mass was dissected from nine oysters from each diet and the control sample, keeping the samples below 0 °C on dry ice. Gonad wet weight for each oyster was measured using calibrated Mettler Toledo AB54-S laboratory scales to a precision of ± 1 mg. Gonad tissue was stored at − 80 °C.

### Fatty acid and lipid analyses

#### Reagents

Solvents were purchased from Sigma-Aldrich Ltd (Dorset, UK) of at least HPLC grade and were not purified further. Lipid standards were purchased from Avanti Polar lipids (Alabaster, AL; via Instruchemie, Delfzijl, NL) and used without purification. Consumables were purchased from Sarstedt AG & Co (Leicester, UK) or Wolf Labs (Wolverhampton, UK).

#### Preparation of oyster gonads for extraction of the lipid fraction

The method used was newly developed for the oyster samples. Frozen adipose tissue was dispersed in an aqueous solution of guanidine and thiourea (6 M/1.5 M) ; 20 × w/v) and diluted with methanol (30%) and TBME (10%). The dispersions were freeze-thawed and agitated before extraction of the lipid and glyceride fraction.

#### Extraction of the lipid fraction

The method used for extracting the lipid fraction was described recently^43^. Briefly, the solution of adipose (20 µL, prepared as above) was injected into a well (96w plate, Esslab Plate, 2.4 mL/well, glass-coated) followed by internal standards (150 µL, Mixture of Internal Standards in methanol (see Table S3), water (500 µL) and DMT (500 µL, Dichloromethane, methanol and triethylammonium chloride, 3:1:0·005). The mixture was agitated (96 channel pipette) before being centrifuged (3.2×*g*, 2 min). A portion of the organic solution (20 µL) was transferred to an analytical plate (96w, glass-coated, Esslab Plate) before being dried (N_2_ (g)). The dried films were re-dissolved (TBME, 30 µL/well) and diluted with a stock mixture of alcohols and ammonium acetate (100 µL/well; propan-2-ol: methanol, 2:1; CH_3_COO·NH_4_ 7.5 mM). The analytical plate was heat-sealed and run immediately.

#### Mass spectrometry

In order to survey the profiles of glycerides, phospholipids and the fatty acids within phospholipids, we used a direct infusion mass spectrometry method developed recently^43,44^ that was based on one that has been used in several studies of samples (infant formula^43^^,^ human dried blood spots^45–48^, plasma^49,50^ and serum^51^. The method consists of positive ion mode, negative ion mode and a negative ion mode with collision-induced dissociation. Positive mode processing used a deviations threshold of 9 ppm and a signal strength threshold of 2. Abundance/signal intensity was plotted using 25, 50, 100% quality control (QC) samples and a correlation threshold of 0.75 was used. Variables with 0% values across all samples were removed before the intensities were signal-corrected. Negative mode processing used a deviations threshold of 9 ppm. QC samples and a correlation threshold of 0.75 was used. A deviations threshold of 12.5 ppm was used for processing of the negative ionization mode with CID, on a list of fatty acids of chain length 14 to 36 with up to six olefin bonds and/or one hydroxyl group. All signals stronger than noise were carried forward. Signals consistent with fatty acids were found in 3/3 samples checked. QC samples consisted of a pooled mixture of aliquots of six samples from across the study. QC concentrations of 0.25, 0.5 and 1.0 were used to assess the correlation between sample concentration and signal intensity.

### Sexual maturation analyses

To assess sexual maturation all gonadal tissue was dissected from 5 oysters from each diet and the control. Tissue was fixed, sectioned, stained using hematoxylin and eosin, and imaged under light microscopy following a standard protocol^52^.

### Data processing and statistical analyses

Power analyses were performed using G*Power (2018, Heinrich-Heine-Universität Düsseldorf, Germany) before experiments started, to ensure experimental designs and sample sizes were appropriate. For the lipid analyses Principal Component Analyses (PCAs) were carried out using Metaboanalyst 4.0^53^ to identify which samples grouped together in a data-driven manner. To account for the difference in gonad mass between samples, abundance values from mass spectrometry data for fatty acids and lipids were scaled by gonad wet weight (Table S4). Statistical analyses of the data were then performed using R Statistics^54^. For the gonad weight analysis ANOVA with post-hoc Tukey's tests were used to compare gonad weight between the diets and control. For the fatty acid and lipid analyses ANOVA with post-hoc Tukey's test and a Holm-Bonferroni correction for multiple comparisons were used to assess differences in the abundance of each fatty acid or lipid between diets, relative to the control^55^. Molview (2015, Herman Bergwerf) was used to generate molecular images for Fig. 3.

## Supplementary information

Supplementary Legends.

Supplementary Data S1.

Supplementary Table S1a.

Supplementary Table S1b.

Supplementary Table S2.

Supplementary Table S3.

Supplementary Table S4.

## Data Availability

All data is available in the manuscript or supplementary materials.
