# Supplementary material for: Microencapsulated algal feeds as a sustainable replacement diet for broodstock in commercial bivalve aquaculture
Source: Sci Rep. 2020 Jul 31;10:12577. doi: 10.1038/s41598-020-69645-0 (PMC7395148; doi:10.1038/s41598-020-69645-0)
Supplement: Supplementary file 1 — Supplementary Legends. [file 41598_2020_69645_MOESM1_ESM.docx]

Supplementary Information for

**Microencapsulated algal feeds as a sustainable replacement diet for broodstock in commercial bivalve aquaculture**

David F. Willer, Samuel Furse and David C. Aldridge

David F. Willer

Email: [dw460@cam.ac.uk](mailto:dw460@cam.ac.uk)

**Supplementary Materials Guide**

- **Data S1:** This document contains a tabulated version of the data used to produce Figure 1, alongside data sources used and assumption made during the calculations
- **Table S1:** Difference in abundance of fatty acids (a) and other lipids (b) in the gonads of oysters fed algae, microcapsules, or algae + microcapsules over a six-week conditioning period, relative to pre-conditioning controls. Abundance was calculated from mass spectrometry data using total wet gonad weight as a scaling factor. n = 9 oysters per diet. Significance ratings: * * * *p* < 0.001, * * *p* < 0.01, * *p* < 0.05, ns *p* > 0.05. Holm-Bonferroni method used to correct for multiple comparisons. For (a) all analysed fatty acids are presented, for (b) only the lipids with a significant difference between diets are presented and the remainder are in table S2.
- **Table S2:** Difference in abundance of all analysed lipids in the gonads of oysters fed algae, microcapsules, or algae + microcapsules, relative to control samples. Abundance values were calculated from mass spectrometry data using total gonad wet weight as a scaling factor. n = 9 oysters per diet. Significance ratings: * * * *p* < 0.001, * * *p* < 0.01, * *p* < 0.05, ns *p* > 0.05. Holm-Bonferroni method used to correct for multiple comparisons.
- **Table S3:** Mixture of external standards in methanol used for extraction of lipid fraction
- **Table S4:** Mass spectrometry spreadsheet for all lipids and fatty acids. Tables include calculations to scale abundance values by gonad weight, and calculate the difference between experimental treatments and controls, for all positive mode lipids, negative mode lipids, and fatty acids.
