# Supplementary material for: Microencapsulated algal feeds as a sustainable replacement diet for broodstock in commercial bivalve aquaculture
Source: Sci Rep. 2020 Jul 31;10:12577. doi: 10.1038/s41598-020-69645-0 (PMC7395148; doi:10.1038/s41598-020-69645-0)
Supplement: Supplementary file 2 — Supplementary Data S1. [file 41598_2020_69645_MOESM2_ESM.docx]

**Supplementary Data S1**

This document contains a tabulated version of the data used to produce Figure 1, alongside data sources used and assumption made during the calculations

| **Feed** | **CO2 emissions**  **(kg CO_2_ eq / MJ digestible energy)** | **Energy footprint**  **(MJ used / MJ digestible energy)** | **Cost**  **($ USD / MJ digestible energy)** |
| --- | --- | --- | --- |
| Photobioreactor live algae | 0.0800 | 1.35 | 0.333 |
| Hatchery live algae | 2.67 | 45.0 | 11.2 |
| Microencapsulated algae complete | 0.0382 | 0.321 | 0.0386 |
| Microcapsule component: heterotrophic algae | 0.0450 | 0.600 | 0.0570 |
| Microcapsule component: recycled vegetable oil | 0.00186 | 0.0164 | 0.00595 |
| Microcapsule component: sustainably sourced carnauba wax | 0.0757 | 0.0810 | 0.0416 |

Photobioreactor algae

- This refers to algae grown with optimal light and nutrient inputs on a large industrial scale in a dedicated facility. Data sources for CO_2_ emissions and energy demand ^1^, cost ^2^, digestible energy ^3^.

Hatchery algae

- This refers to algae grown under conditions typical of a relatively efficient bivalve hatchery. Assumes algae produced here are of the same high nutritional quality and hence contain the same level of digestible energy as photobioreactor algae ^3^. Energy demand calculated based on knowledge 20% of hatchery running costs are for electricity to rear algae ^4^. CO_2_ emissions calculated assuming same ratio of energy demand: CO_2_ emissions as for photobioreactor algae ^1^. Cost from ^5,6^.

Microencapsulated diet

- This refers to the microencapsulated diet used during our investigation, consisting of 50% *Schizochytrium* algae grown heterotrophically on food waste, and a proprietary encapsulant and non-ionic surfactant consisting primarily of recycled vegetable oil (30%) and sustainably sourced carnauba wax (20%). Calculations use data indicating vegetable oils and wax in microcapsule encapsulant are digestible by bivalves and assumes encapsulant consists solely of lipids ^3,7,8^. Data sources for CO_2_ emissions and energy demand ^1,9,10^, cost ^2,9,10^, digestible energy ^11^.

References

1. Azari, A., Noorpoor, A. R. & Bozorg-Haddad, O. Carbon footprint analyses of microalgae cultivation systems under autotrophic and heterotrophic conditions. *Int. J. Environ. Sci. Technol.* **16**, 6671–6684 (2019).

2. Abayomi, O., Tampier, M. & Bibeau, E. Microalgae technologies & processes for biofuels / bioenergy production in British Columbia: Current technology, Suitability & Barriers to Implementation. *Seed Sci.* **January**, (2009).

3. Willer, D. F. & Aldridge, D. C. Microencapsulated diets to improve growth and survivorship in juvenile European flat oysters ( Ostrea edulis ). *Aquaculture* **505**, 256–262 (2019).

4. Myers, J. A. & Boisvert, R. N. The economics of producing algae and bivalve seed in hatcheries. *Aquaculture* **86**, 163–179 (1990).

5. Gui, Y., Zamora, L., Dunphy, B. J. & Jeffs, A. G. Evaluation of the formulated diet MySpat for feeding hatchery-reared spat of the green-lipped mussel, Perna canaliculus (Gmelin, 1791). *Aquac. Res.* **47**, 3907–3912 (2016).

6. Gui, Y., Kaspar, H. F., Zamora, L. N., Dunphy, B. J. & Jeffs, A. G. Capture efficiency of artificial food particles of post-settlement juveniles of the Greenshell^TM^ mussel, Perna canaliculus. *Aquac. Res.* **464**, 1–7 (2016).

7. Aldridge, D. C., Elliott, P. & Moggridge, G. D. Microencapsulated BioBullets for the control of biofouling zebra mussels. *Environ. Sci. Technol.* **40**, 975–979 (2006).

8. Willer, D. & Aldridge, D. C. Microencapsulated diets to improve bivalve shellfish aquaculture. *R. Soc. Open Sci.* **4**, 171142 (2017).

9. Li, H. *et al.* Determination of Carbon Footprint using LCA Method for Straight Used Cooking Oil as a Fuel in HGVs. *SAE Int. J. Fuels Lubr.* **7**, 623–630 (2014).

10. Reijnders, L. & Huijbregts, M. A. J. Palm oil and the emission of carbon-based greenhouse gases. *J. Clean. Prod.* **16**, 477–482 (2008).

11. Ramos-Vega, A., Rosales-Mendoza, S., Bañuelos-Hernández, B. & Angulo, C. Prospects on the Use of Schizochytrium sp. to Develop Oral Vaccines. *Frontiers in Microbiology* **9**, 2506 (2018).
